# Online selfie behavior and consideration of cosmetic surgery in teenage girls: The mediating roles of appearance comparisons and body dissatisfaction

**DOI:** 10.1371/journal.pone.0318245

**Published:** 2025-02-06

**Authors:** Yunduan Li, Hong Chen, Yan Zou, Yanshuo Guo, Liying Gao, Xinyu Xu

**Affiliations:** 1 School of Psychology, Northwest Normal University, Lanzhou, People’s Republic of China; 2 Key Laboratory of Behavioral and Mental Health of Gansu Province, School of Psychology, Northwest Normal University, Lanzhou, People’s Republic of China; 3 Chongqing Vocational Institute of Engineering, Chongqing, China; University of Hyderabad, INDIA

## Abstract

Although cosmetic surgery can repair functional impairments caused by deformities, improve appearance, and enhance self-esteem, it also carries certain risks, complications, and even death. To reduce the risks to life and property caused by repetitive or inappropriate procedures, it is important to identify the prerequisites that lead individuals to pursue cosmetic surgery. Some studies have shown a significant association between selfies and cosmetic surgery consideration, with prior research focusing on offline selfie behaviors rather than the relationship between online selfies (including posting and viewing selfies) and consideration of cosmetic surgery. From the perspective of online selfie behaviors, this study explores the relationship between online selfie behaviors and consideration of cosmetic surgery, as well as its internal mechanisms. The study included 762 teenage girls with an average age of 16.85 years who completed the Online Selfie Behavior, Appearance Comparison, Body Dissatisfaction, and Consideration of Cosmetic Surgery scales. Structural equation modeling (SEM) was used to examine the direct and indirect relationships between posting or viewing selfies and willingness to undergo cosmetic surgery. The results of the current study indicate that viewing selfies on social media, rather than posting them, is a key factor affecting consideration of cosmetic surgery, and this influence occurs directly or indirectly through appearance comparison and body dissatisfaction. This suggests that in the current era of frequent social media activities, a more relaxed social and cultural environment and diverse aesthetic standards are necessary. Parents and educational psychologists should focus on guiding girls to develop positive body image, carefully considering the impact of selfie images on body dissatisfaction in teenage girls on social media, and the resulting demand for beauty products and cosmetic surgery.

## 1. Introduction

The online selfie behavior mainly involves posting (sharing selfies on social media) and viewing (checking out others’ selfies and liking/commenting) selfies on social media [1]. Selfies are one of the primary social media activities among young people currently, with females engaging in selfie behavior more than males. Surveys have found that females under the age of 30 have a higher frequency on image-centered social platforms such as Instagram and TikTok compared to males [2], and they also take and post more selfies than males [3]. Boursier et al. (2020) found that engaging in online selfie behavior can increase appearance anxiety [4], even leading to negative psychological and behavioral effects such as depression, appearance dissatisfaction [5], eating disorders, low self-esteem [6], more targeted body image self-criticism [7], and heightened social body anxiety [8]. In order to address these psychological imbalances, individuals strive harder to manage their online image [9], including posing and editing photos, and even considering cosmetic surgery to alter their appearance [8]. It can be argued that increased online selfie behavior is correlated with a greater consideration for cosmetic surgery [10]. While statistics show an increase in male cosmetic procedures in recent years [11], this change remains relatively small compared to females [12]. From a sociocultural perspective, this is related to the notion held by females that self-esteem and value are closely linked to physical attractiveness [13,14]. Therefore, the phenomenon of female selfie behavior on social media has gradually attracted the attention of researchers. However, previous studies have lacked exploration on whether there are differences in how different online selfie behaviors impact female motivations for cosmetic surgery.

### 1.1. Selfie viewing and posting had different effects on consideration of cosmetic surgery

Although many previous studies have confirmed a strong correlation between online selfie behavior and cosmetic surgery [1,15]. But, further research on this topic still needs to be enhanced. On the one hand, although some studies have discussed the difference in the impact of selfie posting and viewing on individuals’ body image, they have not delved into the mechanism of this difference and whether it affects their cosmetic surgery consideration. For example, research by Wang et al. (2019) shows that the act of viewing selfies, rather than posting selfies, has a significant impact on individuals’ facial dissatisfaction, but it does not explain the possible internal mechanism and external influencing factors that cause the difference between the two [16]. Based on previous studies, we speculate this may be related to the fact that posting always occurs at the peak of self-experience [17,18]. On the other hand, most studies have focused on adult female populations, primarily exploring their attitudes or behaviors towards cosmetic surgery [19]. However, there is limited research on the consideration of teenagers in the early stages of self-esteem development to undergo cosmetic surgery, which hinders the ability to predict and prevent the harmful effects of cosmetic surgery. So, the first question we examined was whether the act of posting and viewing selfies were strongly correlated with consideration of cosmetic surgery, respectively among teenage girls. We hypothesized that there would be significant association between posting selfies and cosmetic surgery consideration, and also between viewing selfies and consideration of cosmetic surgery (H1).

### 1.2. The mediating role of appearance comparison and body dissatisfaction between online selfie behavior and consideration of cosmetic surgery

Previous studies have found that body dissatisfaction is the primary reason individuals choose to have cosmetic surgery [20,21]. The Tripartite Influence Model suggests that appearance pressure is conveyed through social information, which triggers unrealistic appearance ideals [12]. In other words, when a girl’s body image does not meet the societal standards for body image in which she lives, conflicts between self-image and external environment demands are likely to arise, leading to self-discrepancy. Self-discrepancy, which refers to the inconsistency between a person’s self-perception and their ideal self-perception [22], is a significant factor in driving individuals to address this inconsistency [23]. When individuals with a significant self-discrepancy perceive the ideal slim image, they are more likely to experience heightened body dissatisfaction [24] and to opt for cosmetic surgery [25]. The state in which the female ideal body image deviates from the actual body shape has become a common phenomenon in various societal and cultural environments, laying the environmental foundation for the body dissatisfaction of many girls [26].

Tripartite Influence Model attributes factors influencing individuals’ body image perception to three aspects: family, peers, and media, which directly or indirectly impact our body evaluations, promote the internalization of aesthetic norms, and comparison with others’ appearance [27]. Studies have found that perceptual pressures from the media are significantly stronger than those from peers, family, health, or beauty professionals when it comes to the decision to undergo cosmetic surgery and use beauty products [28]. Social media plays a foundational role in amplifying these comparisons [29], as Kidd et al. (2024) have for the first time incorporated social media into the Elaborated Sociocultural Model that pertains to the establishment of individual body image within the current sociocultural environment [2]. This model highlights the significant role of social media in body image construction. Firstly, unlike traditional media’s one-way information dissemination, social media allows users to actively engage in information dissemination with minimal restrictions, even in the production process [2]. Secondly, the social media selfie craze has increased our exposure to mainstream beauty standards and reinforced the prevailing discourse on beauty [30]. Thirdly, the openness and speed of information dissemination on the Internet enable individuals to access a wider range of comparable entities, such as strangers or celebrities.

According to social comparison theory, individuals tend to compare themselves to others who are better off in social comparisons, known as upward social comparisons [31]. In social media activities, individuals who internalize a slim ideal compare themselves more frequently to peers and idealized media images, that is, comparing themselves to those who are perceived as thinner and more attractive [32,33]. On one hand, such comparisons can lead to negative outcomes, triggering concerns about body image and the pursuit of change [2]. On the other hand, upward social comparison can also motivate individuals to continuously approach the comparison target [34]. Therefore, regardless of whether the comparison results in anxiety or motivation, it ultimately prompts individuals to actively seek changes to narrow the “beauty” gap with the comparison target [35]. For example, research has shown that viewing celebrities’ selfies is more influential in shaping attitudes toward cosmetic surgery than viewing selfies of their peers [36].

From the above explanation, it is important to consider body dissatisfaction as an internal individual potential risk factor for cosmetic surgery, while appearance comparison is an environmental interaction factor. However, it is unclear whether comparing one’s appearance and feeling dissatisfied with one’s body mediate the relationship between online selfie behavior and consideration of cosmetic surgery. Therefore, the second question of our study was: Is there a potential mechanistic difference in the impact of posting and viewing selfies on consideration of cosmetic surgery? We hypothesize that selfie viewing can indirectly affect consideration of cosmetic surgery by body dissatisfaction or appearance comparison, it can also influence individual consideration of cosmetic surgery through the chain mediating effect of appearance comparison leading to body dissatisfaction (H2). While selfie posting does not directly or indirectly effect consideration of cosmetic surgery (H3). The direct effect of the two kinds of online selfie behavior on consideration of cosmetic surgery was not significant (H4).

### 1.3. The present study

**Fig 1:**
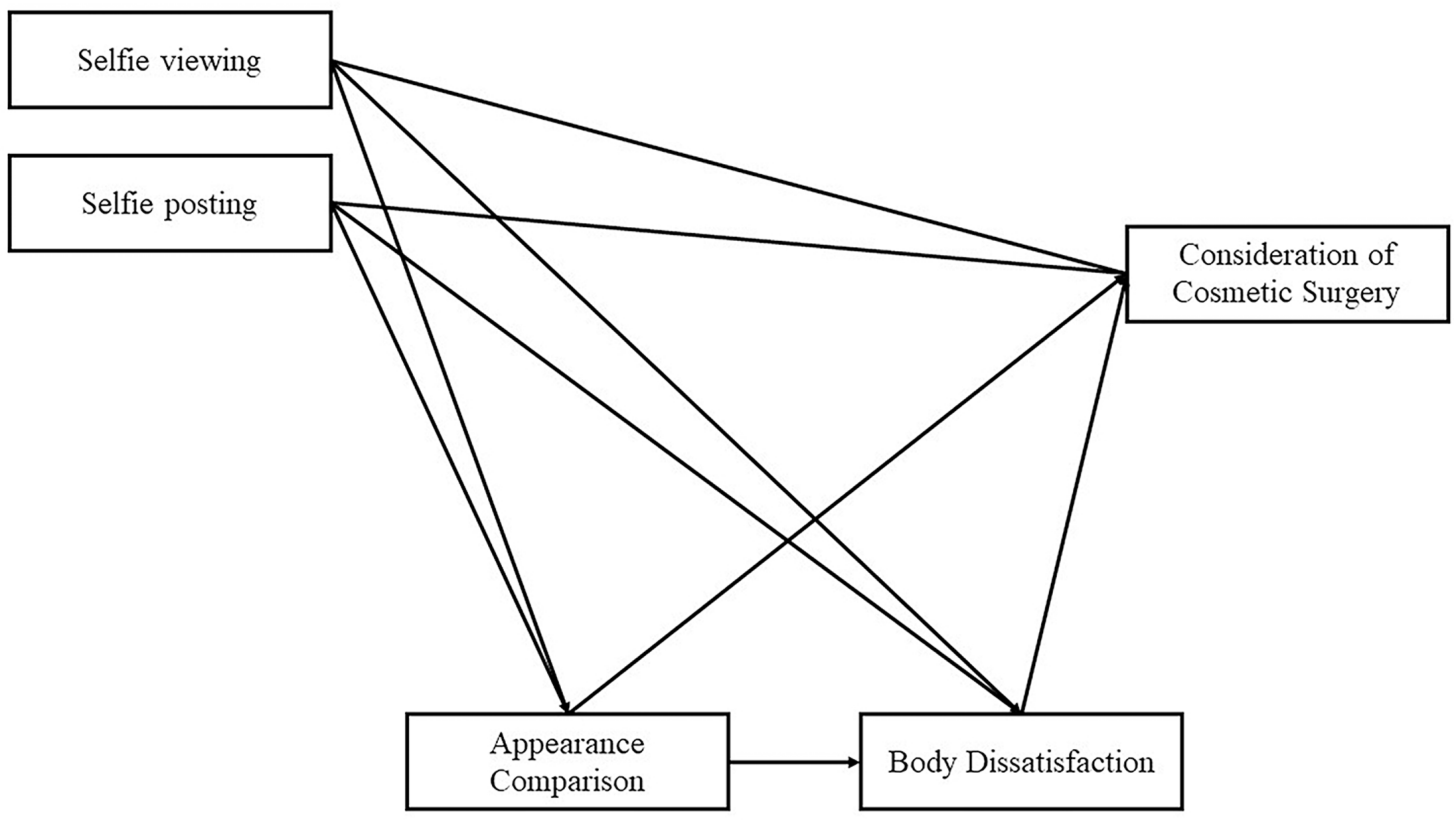
Theoretical hypothesis model of chain mediation.

In general, the selfie behavior on social media has intensified individuals’ willingness to change their body image through cosmetic surgery. However, research on the differences in the impact of posting and viewing selfies on the consideration of cosmetic surgery and their underlying mechanisms is limited. This is particularly important for teenagers who are in a rapid developing self-awareness stage, as their perception and evaluation of their body image not only influence their desire for cosmetic surgery, but also pose a threat to their self-awareness development. From the perspective of self-discrepancy and social comparison theory, this study examined the relationship between teenage girls’ online selfie behaviors (i.e., posting and viewing selfies) and their cosmetic surgery consideration. The study analyzed the direct and indirect mediating effects of appearance comparison and body dissatisfaction. We hypothesize that there will be an indirect pathway from engaging in online selfie behaviors to cosmetic surgery consideration, mediated by appearance comparison and body dissatisfaction, as illustrated in Fig 1, which serves as the theoretical hypothesis model. Furthermore, based on the theoretical connections mentioned earlier, we anticipate an indirect association between selfie posting or viewing and cosmetic surgery consideration, also mediated by appearance comparison and body dissatisfaction (in sequence).

## 2. Method

### 2.1. Participants

Convenient sampling technique was used to recruit participants from three schools in Sichuan province and Chongqing municipality, China. Participants voluntarily signed up through campus posters, and those eligible to participate in the study had to be teenage girls who had not undergone any cosmetic alterations (including liposuction, nose reshaping, breast augmentation or reduction, facial implants, ear surgery, eyelid surgery, hair removal, and acne treatments, etc.). The sample consisted of 762 teenage girls aged 15 to 19 years-old (10.4% younger than 15, 0.38% older than 18), with an average age of 16.85 years (SD = 1.052). Urban students accounted for 58.8% and 18.5% came from single-parent families, 24.3% had a monthly household income less than RMB 2,000, and 20.6% had a monthly household income more than RMB 6,000. Participants were asked to complete a study on their own body image and willingness to changing. Prior to the survey, we obtained informed consent from each participant and their parents (parents were informed collectively at the beginning of the school term).

### 2.2. Procedure and measures

#### 2.2.1. Procedure.

Participants were asked to anonymously fill out a Chinese electronic questionnaire on the Wenjuanxing platform (a platform that provides the equivalent of Amazon Mechanical Turk), with no requirement to provide any personal identity information. It takes about 20 minutes to complete the questionnaire. The measurements were presented to all participants in the following order: demographic items, appearance comparisons, body dissatisfaction, selfie behaviors, and consideration of cosmetic surgery. All participants were informed about the study’s aims and that the results would be used for research purposes, and then they submitted their informed consent online before completing the questionnaire. Researchers were present throughout the survey, and participants could ask them questions. After completing the survey, participants were eligible to apply for additional course credits. It started on March 19 and ended on March 30, 2022. Our study was approved by the Ethics Committee of the School of Psychology, Northwest Normal University (Reference No: 2022046).

### 2.3. Measures

**Online selfie behavior:** We used two scales to measure selfie viewing [37] and selfie posting [38] separately, adapted from the Negative Social Media Use Scale by Frison (2016) and the Photo-Related Behavior Scale by Meier (2014). Both of them revised by Luo (2017) and has good reliability and validity in the Chinese sample [39]. The selfie viewing intensity scale consists of 3 items, including “Do you often browse selfies posted by others on WeChat/Weibo/QQ?” rated on a 5-point scale, with higher average scores indicating higher frequency of browsing others’ selfies. The selfie posting scale also includes 3 items, such as “Do you often post selfies on WeChat/Weibo/QQ?” rated on a 5-point scale, with higher average scores representing more frequent posting of selfies. All items in the measures require participants to answer based on their experiences in the past 30 days, and the mean scores of both subscales were included in the data analysis. These modified subscales have been previously used in Chinese adolescent populations, with reported Cronbach’s α reliability coefficients of 0.61 and 0.76 [39]. The Cronbach’s α in the current study are 0.78 and 0.73.

**Appearance comparison:** The appearance comparison scale consists of The Physical Appearance Comparison Scale (PACS) (Chinese version) revised by Chen Hong (2007) [40] and the Body Comparison Scale compiled by Thompson (1998) [1], reflecting individual traits related to social comparisons of body appearance. Each scale has 5 items, making a total of 10 items in the entire survey tool, with all items scored on a 5-point scale, such as “I compare how I dress to how others dress at parties or other social occasions” and “I compare my weight to the weight of others when I am with them”. 1 represents “never” and 5 represents “always”, with the 4th item being a reverse-scored item. The higher the average score, the more frequent the appearance comparisons. The questionnaire requires participants to answer based on their experiences in the past 30 days. Past research has shown that these two tools have good reliability and validity, with the internal consistency coefficient of the Chinese version questionnaire being 0.85 [40], and the Cronbach’s alpha of this study is 0.94.

**Body dissatisfaction:** The body dissatisfaction was measured using the Negative Physical Self-Scale-Appearance scale (NPSS-A) [41]. This subscale consists of 11 items to assess thoughts, emotions, projections, and behaviors related to dissatisfaction with appearance (e.g., “I feel discouraged about my face”). Participants rated each item on a 5-point Likert scale ranging from 1 (never liked myself) to 5 (always liked myself). They were asked to answer based on their experiences in the past 30 days, and the average score of all items was calculated as the final statistical analysis indicator, with higher scores indicating lower body satisfaction. The scale has reported good reliability, stability, and validity in Chinese adolescent samples, with a Cronbach’s alpha coefficient of 0.87 [41]. The reliability coefficient Cronbach’s α in this study was 0.93.

**Consideration of cosmetic surgery:** We assessed girls’ willingness to consider cosmetic surgery through the five “Consideration” subscales of Willing to Have Cosmetic Surgery Scale (WHCSS) [42], consisting of 5 items reflecting the extent to which respondents may consider undergoing cosmetic surgery in the future. For example, “In the future, I may consider some form of cosmetic surgery” and “If you could get a plastic surgeon to change your appearance for free, would you?” Participants were asked to respond to each item based on their experiences in the past 30 days, with all items scored on a 7-point Likert scale ranging from 1 = strongly disagree to 7 = strongly agree. The average score across all items was used for data analysis, with higher scores indicating a stronger willingness to use cosmetic surgery as a means to change appearance. Jackson and Chen (2015) validated the reliability and validity of this subscale in samples of adult and adolescent females in China, with Cronbach’s alphas of 0.94 and 0.86, respectively [12]. The Cronbach’s for the current sample was 0.75.

### 2.4. Analysis methods

#### 2.4.1. Demographics.

Participants provided demographic information such as age, monthly household income and origin of student. Monthly household income and origin of student (0: rural, 1: urban) were used to measure the socio-economic status of the subjects. Monthly household income is divided into 9 grades, with a score of 1 to 9, the higher the score, the higher the amount of monthly household income.

#### 2.4.2. Data analysis.

The bivariate correlation between variables was tested using SPSS 27.0. Structural equation model (SEM) and Mplus 8.3 software were used for maximum likelihood estimation to investigate the influence of selfie posting and selfie viewing on cosmetic surgery intention. All variables in this model are observed, and the average score of each scale item is calculated as the observed value. The selfie behavior of the independent variable calculates the average score of questions 1–3 in the scale to get the selfie viewing score, and the average score of questions 4–5 to get the selfie posting score, so as to reduce the complexity of the model. The model also takes age, monthly household income and place of origin as covariables. The bias correction bootstrap method (5000 times) was used to calculate the 95% confidence interval and test the indirect effect. If the 95% confidence interval is not zero, the indirect effect is considered significant [43]. Since the variables in this model are all observed variables, the model is a full model [44].

## 3. Results

### 3.1. Common methods bias analyses

Because the data were collected from questionnaires, common method deviation might occur. To check and test common method bias, Harman’s single-factor test using confirmatory factor analysis was conducted. The present study conducted a factor analysis on all items of SBS、ACS、NPSS-A and WHCSS and extracted a common factor from these items. Exploratory factor analysis results show that there are 6 factors with eigenvalues greater than 1. And the interpretation rate of the first factor was 40.11%, less than 50%. The results indicated that there was no common method bias in the questionnaires used in this study [45,46].

### 3.2. Descriptive statistics and correlation analysis

Table 1 shows descriptive statistics of the surveyed variables. No measurements exhibited significant ceiling or floor effects. Skewness values were below 2, indicating a general normality of the data [47].

**Table 1:** Min score, Max score, Means, SDs, and Skewness among study variables.

	Min	Max	Mean	SD	Skewness
Age	14.00	21.00	16.85	1.05	0.04
MHI	1.00	3.00	1.96	0.67	0.04
SV	1.00	5.00	2.43	0.93	0.44
SP	1.00	4.00	1.69	0.63	0.94
AC	1.00	5.00	1.88	0.78	0.79
BD	1.00	5.00	2.03	0.76	0.51
CSC	1.00	7.00	2.17	1.27	1.06

*Note*. Hereafter, MHI = Monthly household income, SV = Selfie viewing, SP = Selfie posting, AC = Appearance comparison, BD = Body dissatisfaction awareness, CSC = Cosmetic surgery consideration.

Table 2 shows the bivariate correlations between variables. From the results, it can be seen that there is a significant positive correlation between selfie viewing and selfie posting, appearance comparison, body dissatisfaction, and consideration of plastic surgery (0.246 <  *r* < 0.510, *p*s < 0.001). There is also a significant positive correlation between selfie posting and appearance comparison, body dissatisfaction, and consideration of cosmetic surgery (0.113 < *r* < 0.192, *p*s < 0.001). There is a significant positive correlation between appearance comparison and body dissatisfaction, and consideration of cosmetic surgery (0.446 < *r* < 0.668, *p*s < 0.001). These are consistent with our hypothesis, and show that the more selfie behaviors a girl has, the stronger body dissatisfaction and intention to undergo cosmetic surgery in the future. Moreover, age, a control variable, does not show significant correlation with other variables. There is a significant positive correlation between student origin (OS) and monthly family income (MHI) and selfie viewing (0.088 < *r* < 0.221, *p*s < 0.05), and between monthly family income and selfie viewing and consideration of cosmetic surgery (0.121 < *r* < 0.169, *p* < 0.05). This can be explained as girls from urban families or with higher family income having more online selfie viewing behavior and stronger intentions to undergo cosmetic surgery in the future. Therefore, in the mediational analysis of structural equation model, we will include OS and MHI as control variables.

**Table 2:** Bivariate correlations among study variables.

	Age	OS	MHI	SV	SP	AC	BD	CSC
Age	–							
OS	−.094**	–						
MHI	−0.01	.221***	–					
SV	−0.01	.088 *	.169***	–				
SP	0.04	−0.01	0.06	.246***	–			
AC	−0.01	0.05	0.06	.510***	.192***	–		
BD	−0.05	−0.03	0.03	.466***	.170***	.668***	–	
CSC	0.01	0.02	.121**	.337***	.113**	.446***	.494***	–

*Note.* Hereafter, OS = Origin of student.

### 3.3. Mediation analysis

As mentioned earlier, the more online selfie behaviors, the higher frequency of appearance comparisons, but there is no clear delineation of the causal relationship between the two. Therefore, in this study, we examined two models. Model 1 uses selfie behaviors as the independent variables, as shown in Fig 1, while Model 2 uses appearance comparison as the independent variable and selfie behaviors as the mediating variables respectively. After fitting the data to the two models, it was found that the fit indices for Model 2 were significantly worse than those for Model 1 (see Table 3). Therefore, Model 1 with selfie behavior as the independent variable is the preferred choice. The results of the standardized path coefficients in the structural equation model indicate that Model 1 is a full model.

**Table 3:** Chain mediation effects comparison of Model 1 and Model 2.

Model	AIC	BIC	RMSEA	CFI	TLI	SRMR
Model 1	5110.512	5207.867	0.000	1.000	1.000	0.000
Model 2	6791.047	6906.946	0.169	0.979	0.621	0.029

**Fig 2:**
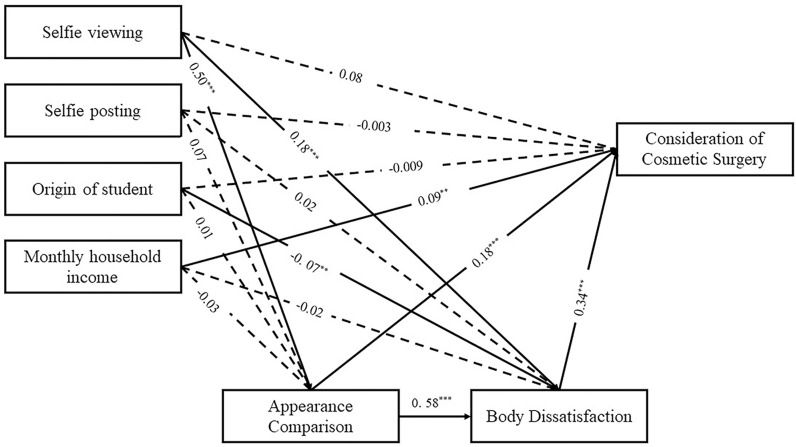
Result model of chain mediation.

As shown in Fig 2, this figure illustrates the chain-mediated result model. Table 4, first, appearance comparison (β = 0.09, BC 95% CI [0.51, 0.20]) and body dissatisfaction (β = 0.05, BC 95% CI [0.06, 0.13]) mediated the relationship between selfie viewing and consideration of cosmetic surgery. In addition, the chain mediation analysis showed that selfie viewing had significant indirect effects on appearance comparison, body dissatisfaction and consideration of cosmetic surgery (β = 0.10, BC 95%CI [0.09, 0.19]), while selfie viewing had no significant direct effects on consideration of cosmetic surgery (β = 0.08, SE = 0.04, *p* = 0.06). Second, appearance comparison (β = 0.01, BC 95% CI [0.51, 0.20]) and body dissatisfaction (β = 0.005, BC 95% CI [0.06, 0.13]) had no significant mediating effect between selfie posting and consideration of cosmetic surgery. Chain mediation analysis showed that selfie posting had no significant indirect effect on appearance comparison, body dissatisfaction and cosmetic surgery intention (β = 0.01, BC 95%CI [0.00, 0.06]), while selfie posting had no significant direct effect on consideration of cosmetic surgery (β = −0.003, SE = 0.03, *p* = 0.93). Path analysis further examined the relationship between appearance comparison and body dissatisfaction. The results showed that appearance comparison had a significant effect on body dissatisfaction (β = 0.58, *p* < 0.001).

**Table 4:** Chain mediation effects of online selfie behavior on consideration of cosmetic surgery via appearance comparison and body dissatisfaction.

Effects	β	SE	*p*	BC 95% CI	
				Lower	Upper
Direct effects					
SV→CSC	0.08	0.04	0.06	–	–
SV→AC	0.50	0.03	<0.001	–	–
SV→BD	0.18	0.04	<0.001	–	–
SP→CSC	−0.003	0.03	0.93	–	–
SP→AC	0.07	0.04	0.06	–	–
SP→BD	0.02	0.03	0.58	–	–
AC→CSC	0.18	0.05	<0.001	–	–
BD→CSC	0.34	0.05	<0.001	–	–
AC→BD	0.58	0.03	<0.001	–	–
Indirect effects					
SV→AC→CSC	0.09	0.03	–	0.04	0.14
SV→BD→CSC	0.06	0.01	–	0.04	0.09
SV→AC→BD→CSC	0.10	0.02	–	0.07	0.13
SP→AC→CSC	0.01	0.01	–	0.001	0.029
SP→BD→CSC	0.01	0.01	–	−0.02	0.02
SP→AC→BD→CSC	0.01	0.03	–	0.00	0.03

In summary, after controlling for covariates, we found that appearance comparison and body dissatisfaction were mediators of consideration of cosmetic surgery in selfie viewing [48]. However, appearance comparison and body dissatisfaction had no significant direct and indirect mediating effects between selfie posting and consideration of cosmetic surgery. Participants who viewed more selfies reported higher levels of appearance comparison and body dissatisfaction, which increased their desire to have cosmetic surgery, the influence of selfie viewing on consideration of cosmetic surgery was mainly caused by body dissatisfaction intermediaries (47.40%), and appearance comparison intermediaries accounted for 26.60%. Among selfie viewing and consideration of cosmetic surgery, the chain mediation effect of appearance comparison and body dissatisfaction accounted for 28.20%.

After testing the significance of the mediating effects of the model, we found that selfie viewing could significantly affect the consideration of teenage girls’ consideration of cosmetic surgery through three paths: appearance comparison and body dissatisfaction as simple mediators, and the chain mediation between selfie posting and body dissatisfaction. In order to investigate whether the significant differences of the mediating effects in these three paths, and to determine which of the two mediating variables in selfie viewing has a greater impact on the consideration of cosmetic surgery, we further conducted a comparison test of the mediating effects in Model 1 with selfie viewing as the independent variable. As we can see Table 5, it can be seen that among the three paths with significant mediating effects between selfie viewing and consideration of cosmetic surgery, the chain mediation effect of selfie viewing through appearance comparison and body dissatisfaction has the largest mediating effect size, accounting for 29.4% of the total mediating effect. And was also found that the chain mediation effect through appearance comparison and body dissatisfaction was significantly stronger than the simple mediation effect of body dissatisfaction (β = 0.058, BC 95% CI [−0.119, −0.013]), while there was no significant difference compared to the simple mediation effect of appearance comparison. From this, we can infer that compared to body dissatisfaction, appearance comparison is a more important mediating variable in the influence of selfie viewing on the consideration of cosmetic surgery.

**Table 5:** Mediation effects comparison of online selfie viewing on consideration of cosmetic surgery via appearance comparison and body dissatisfaction.

Comparison of mediating effects	Estimate	Lower 2.5%	Upper 2.5%
ind1：SV→AC→CSC	0.122	0.052	0.2
ind2：SV→BD→CSC	0.077	0.046	0.121
ind3：SV→AC→BD→CSC	0.135	0.093	0.189
Total mediation effect	0.458	0.361	0.554
ind1/total	0.267	0.118	0.436
ind2/total	0.167	0.096	0.279
ind3/total	0.294	0.192	0.458
ind1-ind2	0.045	−0.052	0.142
ind1-ind3	−0.012	−0.114	0.087
**ind2-ind3**	−**0.058**	−**0.119**	−**0.013**

## 4. General discussion

Our study constructs a chain mediation model with appearance comparison and body dissatisfaction as mediating variables from the perspective of individual-environment interaction. Furthermore, we demonstrated that the Elaborated Sociocultural Model can be utilized to explain the mediating role of appearance comparison and body dissatisfaction in the influence of social media selfie behavior on teenage girls’ intentions for cosmetic surgery within the cultural context of eastern collectivism society. This explains why selfie viewing significantly influences teenage girls’ consideration of cosmetic surgery (the direct and indirect mediating effects of appearance comparison and body dissatisfaction), but not selfie posting. However, our results found that appearance comparison had a marginal significant mediating effect between posting selfies and considering cosmetic surgery. This supports the findings of Thomas et al. (2017), who suggest that when people are happy with their self-image, they post selfies and have a short-term boost in self-esteem [18]. But the high self-esteem at this moment did not last, but was undermined by the delay. However, over time, the likes, comments and viewing others’ selfies can affect her satisfaction with her appearance, causing her to want to change. Our findings once again confirm the impact of environmental factors such as family, peers, and media in shaping body image as proposed in the Tripartite Influence Model [49].

The results of this study suggest that not all online selfies affect an individual’s desire to have cosmetic surgery. Specifically, we found that viewing selfies can directly or indirectly influence cosmetic surgery considerations (through appearance comparison and body dissatisfaction). The appearance comparison apparently plays a significant mediating role in this relationship, which is consistent with the findings of previous studies on Chinese adult women [50,51]. Our study builds on previous research that included teenage girls aged 15 to 19 years. Body dissatisfaction was also identified as an internal risk factor for the link between viewing selfies and consideration of cosmetic surgery. In addition, appearance comparison and body dissatisfaction may have independently contributed to the association between selfie viewing and consideration of cosmetic surgery. These findings suggest that selfies viewing online is an important behavioral risk factor for cosmetic surgery. This expands our understanding of how selfies are a disruptive set of factors that affect appearance perception, in line with differing perspectives brought about by media’s expansion of social comparisons.

Our study revealed that body dissatisfaction mediated the relationship between selfie viewing and consideration of cosmetic surgery, but not between selfie posting and consideration of cosmetic surgery. This finding contradicts previous studies by Jackson and Chen (2015) [52]. This difference may be related to differences in psychological development between the two sample groups. In the study by Jackson & Chen (2015) [52], the sample consisted of male and female undergraduates, whereas our study focused on teenage girls. According to developmental psychology, adolescence marks the beginning stage of self-esteem development. They tend to focus more on their appearance, words, and actions, seeking recognition from others to demonstrate their value [53]. Consequently, they are more vulnerable to the judgment of others and environmental influences, which in turn leads to increased self-doubt and dissatisfaction. This further illustrates the significant role of social comparison in the relationship between teenage girls’ selfie behavior and their consideration of cosmetic surgery.

## 5. Implications

The study examined the relationship between online selfie behaviors (including selfie viewing and posting), appearance comparison, body dissatisfaction, and consideration of cosmetic surgery among teenage girls, revealing for the first time the different pathways by which selfie viewing and selfie posting influence cosmetic consideration. Additionally, while confirming the findings of previous studies, this study also found that selfies does not directly cause body dissatisfaction. Instead, body dissatisfaction could mostly arise through the mediation of social comparison factors. These findings have important theoretical and practical value for understanding and advancing social comparison theory (e.g., finding a key relationship between selfie viewing and body dissatisfaction through appearance comparisons) and self-difference theory (e.g., the effect of selfie viewing on cosmetic surgery through the mediating role of body dissatisfaction rather than selfie posting). This is in line with Kidd et al.‘s (2024) integration of social media into the Elaborated Sociocultural Model, enriching the research findings on how social media influences the construction of self-image among teenage girls in eastern cultural context [2].

Research on this topic is more valuable in exploring the influencing factors at the forefront of cosmetic surgery behavior, which can promote the development of more targeted preventive intervention measures, especially in the study of teenagers, which can enhance their resilience against social cultural pressures, establish diverse aesthetic standards and idealized body images, and construct positive body image and body empathy. Intervention studies have found that social media literacy programs aimed at guiding individuals on how to critically evaluate and analyze media and significant others’ information on body image have positive effects [54]. For example, encouraging teenagers to create and view more body neutral images or videos in social media activities can generate higher levels of positive emotions and fewer upward appearance comparisons [55]. When facing cognitive conflicts about body image, attempting to focus attention on internal states and feelings using psychological methods (e.g., body training, mindfulness, attention training techniques, self-focused attention training) can limit individuals’ body dissatisfaction [7]. Additionally, since families and peers have a strong influence on the construction of individuals’ body image, it is also important to guide individuals on how to critically evaluate and analyze information from peers and significant others about body image. During this process, parents should consciously acknowledge and support children’s body image, guide them to form body empathy, emphasize body functionality while downplaying external beauty standards, and encourage them to construct and internalize diverse ideal body images.

## 6. Limitations and future research directions

It is essential to mention some limitations and future directions in our study. First, height and weight were not used to calculate Body Mass Index (BMI, kg/m2), which is an important limitation of our study, as this prevented us from analyzing girls’ body image perception from a more objective perspective in relation to scientific health standards. Nevertheless, we still consider the results of the study commendable, as the perception of body image encompasses both objective quantified cognition and subjective self-attitudes, with the latter often playing a predominant role in individuals’ body satisfaction [56]. However, this should not overshadow the shortcomings of this study. On the contrary, we propose that the results of this study should be interpreted with caution and unanimously believe that future research should consider BMI as a fundamental factor influencing teenagers’ appearance. In order to researchers can compare whether participants’ body image perception deviates from reality and to assess an important criterion for their psychological well-being.

Second, while our research model idealistically explains how online selfies behavior influences the willingness for cosmetic surgery through appearance comparison, body dissatisfaction, and the chain mediation of both, the complexity of social and individual factors makes it necessary for us to expand the scope of our research. Furthermore, our study only examined whether teenage girls’ consideration of cosmetic surgery, rather than their intentional or actual cosmetic surgery behavior. The cross-sectional study design prevents us from tracking their future actual cosmetic surgery behaviors, thus unable to reveal causal relationships between variables. Therefore, the inferences drawn from the results of this study should be approached with caution. Future research should identify the roles of other factors, and use longitudinal study designs to track them for a longer period to observe the predictive ability of their adolescent cosmetic surgery intentions on future cosmetic surgery behaviors. Additionally, as the proportion and frequency of cosmetic surgery among males continue to rise, future research should also focus on examining the relationship between social media behaviors and cosmetic surgery intentions among adolescent males, in order to validate and expand upon the conclusions of this study.

Finally, body compassion as a more adaptive coping strategy helps individuals construct and maintain positive body image, as well as better adjustment in the face of image threats, to reduce self-criticism and narrow self-discrepancy [7]. It is a method characterized by showing kindness and understanding towards oneself, emphasizing the search for common human experiences, and being mindful of one’s own feelings [7,57,58]. Individuals who exhibit these core features of body compassion are considered to be better able to accept their appearance, recognizing the threat to body image without taking action, and valuing their physical functions [59]. There is a wealth of cross-sectional evidence indicating that body compassion is associated with higher body appreciation, functional appreciation, and body image flexibility [7]. Future intervention research can focus on cultivating body compassion traits in girls.

## 7. Conclusion

In summary, this study demonstrates that the more selfies viewing, the more likely to compare their appearance and are more dissatisfied with their bodies, thus reinforcing the consideration of cosmetic surgery. Interestingly, selfie posting cannot explain the consideration of cosmetic surgery or its connection to body dissatisfaction. Overall, these findings suggest that social media behavior, for example selfie viewing, can influence considerations of cosmetic surgery through personal factors (e.g., body dissatisfaction) and interactions with the environment (e.g., appearance comparison). This expands the potential model for considering cosmetic surgery. It is evident that social media is a double-edged sword, and educators need to assist young people in avoiding its harmful effects.
